# Student perception of pilot interprofessional education and care clinical experiences at dental clinics

**DOI:** 10.5116/ijme.5fc7.7b93

**Published:** 2020-12-26

**Authors:** Se-Lim Oh, Bridgitte Gourley, Cynthia Idzik-Starr

**Affiliations:** 1 University of Maryland, Baltimore School of Dentistry, Maryland, USA; 2 University of Maryland, Baltimore School of Nursing, Maryland, USA

**Keywords:** Student perception, pilot interprofessional education, care clinical experiences, dental clinics

## To the Editor

Health education for each profession has been designed in a way that is discipline-specific.^1^ Dentists and primary care providers (PCPs) possess their own specialized health care fields and have focused on the oral cavity or on the body, respectively. However, clinical studies have suggested that there is a strong relationship between oral disease and systemic conditions.^2^^,^^3^ Systemic conditions affect oral health by modifying host response to bacterial plaque. The oral inflammatory disease may have an impact on systemic health via dissemination of bacterial species, host response factors, or some combination thereof.^2^ A recent retrospective observational study reported that noninvasive periodontal therapy might improve health outcomes in chronic systemic conditions such as type 2 diabetes, rheumatoid arthritis, and cerebral vascular disease.^4^ Therefore, it is critical to build a framework for collaboration between dentists and PCPs in order to deliver holistic care for patients. Faculty can facilitate this by creating opportunities for professional students to work together via interprofessional education (IPE) to improve their understanding of interprofessional care (IPC).

Interprofessional education (IPE) is an important pedagogical approach for students in health care professions,^5^ and is thought to enhance communication skills and collaboration among health care professionals.^6^ Although IPE is introduced in a large-scale event including 700 first-year students from all health care professions at the University of Maryland (UM), Baltimore, there was a lack of interprofessional education and care (IPEC) clinical experiences to bring dental students and family nurse practitioner (FNP) students together. In response to this, we designed and conducted two pilot IPEC projects that brought dental residents/students and FNP students together to assess patient systemic conditions and oral disease at dental clinics over the course of a semester.

While two professional students were engaged together in the two pilot IPEC projects, we measured student perceptions toward the IPEC using the Student Perceptions of Interprofessional Clinical Education-Revised (SPICE-R) instrument.^7^ Student perception is one of the important determinants for student behavior.^8^ In this paper, we share lessons learned from our two pilot IPEC projects.

Our interprofessional education and care (IPEC) team members were comprised of family nurse practitioner (FNP) students, postgraduate dental students at the Advanced General Dentistry (AGD), and senior dental students in the oral and maxillofacial surgery (OMFS) clerkship program, who were under the supervision of faculty members from the two professions. The two pilot IPEC clinical experiences took place at dental clinics. Introductory lectures including an overview of the pilot IPEC projects were provided to participating students from the two professions. Family nurse practitioner (FNP) students rotated at dental clinics one day a week; they were paired with dental students. The assignment for participating FNP students was to obtain a targeted medical history from patients and to assist patients if they needed further evaluation from primary care providers (PCPs). The primary assignment for participating dental students was to observe FNP students while taking the medical history and to provide required dental care to patients.

The first IPEC clinical experience took place at the Advanced General Dentistry (AGD) clinic; 14 first-year FNP students and ten postgraduate (PG) dental students at the AGD in their second semester participated. The target patient population was patients visiting the dental clinic for comprehensive dental care. We found that the mean total score from the first year FNP students was significantly higher than the mean total score from the PG dental students at the AGD in the pre-survey using the SPICE-R.

In the pre-survey from the first IPEC, the first-year NP students strongly agreed that their role was clearly defined, and they clearly understood their role within an interprofessional team. However, the postgraduate dental students at the AGD did not perceive their role to be as clear within an interprofessional team. The first year FNP students strongly agreed that participating in educational experiences with students from another health profession enhances their future ability to work on an interprofessional team, but the PG dental students at the AGD did not strongly appreciate the value of educational experiences with other health professional students. This might be because the postgraduate dental students at the AGD had little opportunity to work with other health care professionals during their undergraduate dental education. While FNP students are exposed to abundant educational experiences within a variety of clinical settings and multiple types of providers throughout their regular curriculum, dental education does not routinely expose students to health care professionals outside of dentistry. In addition, most patients visiting the AGD clinic have their own PCPs, and few have gaps in care. The opportunity for the FNP students to contribute may have been diminished. Little interaction between the postgraduate dental students and the NP students occurred at the AGD. Post-survey could not be obtained.

A study reported that early introduction of IPE was an effective way to enhance the learning experience.^9^ Therefore, we implemented the second IPEC module with oral and maxillofacial surgery (OMFS) senior dental students at the dental urgent care clinic in order to introduce the concept of IPE earlier. Patients visiting the dental urgent care clinic do not often have their own PCPs, and many have unmet medical needs. Taking an accurate medical history from these patients is important since most patients require emergency tooth extractions and many will need additional assistance locating medical care to follow up on their underlying health conditions. Therefore, the second IPEC clinical experience took place at the dental urgent care clinic; 20 oral and maxillofacial surgery (OMFS) clerk senior dental students and ten final-year FNP students participated. The target patient population was patients with dental emergencies.

We found the mean total score of the final year FNP students was higher than that of the OMFS senior dental students in the pre-survey. The mean total score of the final year FNP students toward IPEC was improved after their participation in the second pilot IPEC project, while there was no change in the mean total score of the OMFS senior dental students between pre-IPEC and post-IPEC intervention.

The final-year FNP students might perceive educational experiences from their rotation at dental urgent care clinic as a practical addition for holistic patient care through gaining a richer understanding of dental sequelae and further learning how they can engage with dental providers on behalf of their future patients. However, the OMFS senior dental students were not exposed to other health care settings since the clinical experiences were conducted only within the dental urgent care clinic. While IPEC at the dental urgent care clinic was a new exposure for the NP students, it did not provide any opportunities for the dental students to see how PCPs address dental issues in the context of chronic disease management in PCP office settings.

From our pilot IPEC projects, we learned the FNP student perceptions of the IPEC clinical experiences at dental clinics were more positive than dental student perceptions. FNP student perception was improved further after their participation in the pilot IPEC experience at the dental urgent care clinic. Developing opportunities for dental students to participate in the medical aspects of diagnosis and treatment for chronic systemic conditions may help to improve dental student perception of the IPEC. Identifying opportunities to insert these experiences within the dental curriculum will need to be explored further by faculty.

### Conflict of Interest

The authors declare that they have no conflict of interest.
